# MirSNP, a database of polymorphisms altering miRNA target sites, identifies miRNA-related SNPs in GWAS SNPs and eQTLs

**DOI:** 10.1186/1471-2164-13-661

**Published:** 2012-11-23

**Authors:** Chenxing Liu, Fuquan Zhang, Tingting Li, Ming Lu, Lifang Wang, Weihua Yue, Dai Zhang

**Affiliations:** 1 Institute of Mental Health, Peking University, 51 Hua Yuan Bei Road, Beijing 100191, People’s Republic of China; 2 Key Laboratory of Mental Health, Ministry of Health (Peking University), Beijing, 100191, China; 3 Peking-Tsinghua Center for Life Sciences, Beijing 100871, China; 4 Department of Biomedical Informatics, School of Basic Medical Sciences, Peking University Health Science Center, Beijing, 100191, China; 5 Institute of Systems Biomedicine, School of Basic Medical Sciences, Peking University Health Science Center, Beijing, 100191, China

**Keywords:** microRNA, Single nucleotide polymorphism (SNP), Genome-wide association study (GWAS), Expression quantitative trait loci (eQTLs), MirSNP

## Abstract

**Background:**

Numerous single nucleotide polymorphisms (SNPs) associated with complex diseases have been identified by genome-wide association studies (GWAS) and expression quantitative trait loci (eQTLs) studies. However, few of these SNPs have explicit biological functions. Recent studies indicated that the SNPs within the 3’UTR regions of susceptibility genes could affect complex traits/diseases by affecting the function of miRNAs. These 3’UTR SNPs are functional candidates and therefore of interest to GWAS and eQTL researchers.

**Description:**

We developed a publicly available online database, MirSNP (http://cmbi.bjmu.edu.cn/mirsnp), which is a collection of human SNPs in predicted miRNA-mRNA binding sites. We identified 414,510 SNPs that might affect miRNA-mRNA binding. Annotations were added to these SNPs to predict whether a SNP within the target site would decrease/break or enhance/create an miRNA-mRNA binding site. By applying MirSNP database to three brain eQTL data sets, we identified four unreported SNPs (rs3087822, rs13042, rs1058381, and rs1058398), which might affect miRNA binding and thus affect the expression of their host genes in the brain. We also applied the MirSNP database to our GWAS for schizophrenia: seven predicted miRNA-related SNPs (*p* < 0.0001) were found in the schizophrenia GWAS. Our findings identified the possible functions of these SNP loci, and provide the basis for subsequent functional research.

**Conclusion:**

MirSNP could identify the putative miRNA-related SNPs from GWAS and eQTLs researches and provide the direction for subsequent functional researches.

## Background

MicroRNAs (miRNAs) are small non-coding RNA molecules of ~22 nucleotides that primarily mediate post-transcriptional gene silencing processes in animals [1,2]. MiRNAs inactivate specific mRNAs and interfere with the translation of target proteins [3]. In mammals, miRNAs are predicted to control the activities of ~50% of all protein-coding genes [4]. As key post-transcriptional regulators, miRNAs have an important role in a wide range of biological processes, including cell proliferation, differentiation, apoptosis and metabolism [2,3]. Evidence indicates that miRNAs are also involved in the pathogenesis of complex diseases, such as cancer and mental disorders [4,5].

Complementarity to bases 2-8 of the miRNA (the seed site) is important in miRNA-mRNA binding [6,7]. MiRNAs are key regulators of gene expression; therefore, SNPs in the seed sites of miRNA targets may create, as well as destroy, miRNA binding sites, and further affect phenotypes and disease susceptibility [8]. Identifying these seed-site SNPs could help in the further exploration of the molecular mechanism of gene dysregulation. In addition, genetic variants in miRNA genes may also have important roles by affecting miRNA maturation, which may affect disease susceptibility [8]. Certain polymorphisms in miRNA genes have been found to be associated with various complex diseases, including cancers, mental diseases, cardiomyopathy, and asthma (Additional file 1: Table S1).

GWAS and eQTLs are powerful methods for identifying genetic variants contributing to disease risk and gene expression. In a GWAS of schizophrenia (SCZ), Ripke et al. found the most significant SNP (*p* < 1.6 × 10^-11^) within an intron of a putative primary transcript for hsa-mir-137 and found four other SNPs achieving genome-wide significance that were located in predicted target sites of hsa-mir-137 [9]. It was estimated that more than 50% of the protein-coding genes are regulated by miRNAs, and each miRNA may regulate hundreds of potential targets [10,11]. Taking into account large scale of biological significance shown by miRNAs, miRNA-related SNPs could be important variations leading to traits and diseases. Identifying allele-specific miRNA-mRNA interactions may indicate the functional roles of the SNPs from GWAS and eQTLs that presently lack obvious known function.

To help identifying putative miRNA-related SNPs from researchers’ own GWAS and cis-acting eQTLs data set, we have developed a freely available database, named “MirSNP”, which provides SNPs located in predicted miRNA target sites. This database has minor allele frequency (MAF) and linkage disequilibrium (LD) information for the SNPs and has annotations concerning their creative or disruptive effects on putative target sites. The MirSNP database comprises over 414,510 predicted miRNA-related SNPs, enabling users to identify potential miRNA-related SNPs from their own GWAS or eQTLs data. In this work, we applied MirSNP to our schizophrenia GWAS data and several brain eQTLs data as examples.

## Construction and content

### Data sources

To store the mRNA sequences, miRNA data and SNPs, a local Structured Query Language (SQL) database was built using MySQL software. Human miRNAs were downloaded from miRBase18.0 [12]. For SNP catalogs, four tables from dbSNP135 [13] were used (see URLs). To maximize the consistency between different databases, mRNA sequence files were obtained from NCBI (see URLs) rather than from the UCSC genome browser. In total, 42,733 mRNA sequences and 513,249 SNPs located in the 3’UTRs of mRNA were eligible for subsequent analysis.

### Identifying polymorphisms in pre-miRNA genes

**Figure 1:**
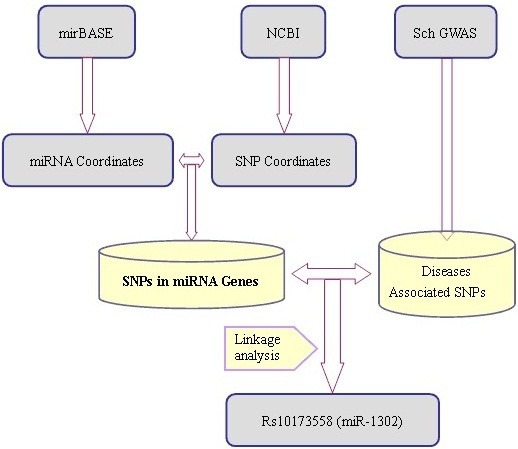
Workflow depicting analysis of variations in/near miRNA genes.

**Figure 2:**
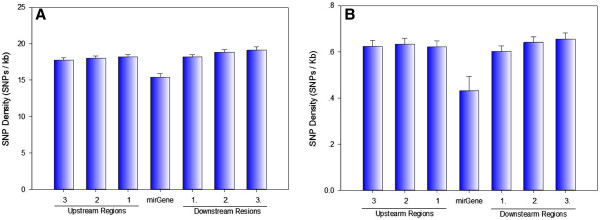
**Density of SNPs in human miRNA genes.** (**A**) SNP density in human pre-miRNAs and flanking regions. Each bar represents the average level of all human miRNAs, error bars represent the standard deviation of the mean value. MiRNA and SNP information came from mirBASE18 and dbSNP135, respectively. Figure A shows the result of all SNPs in dbSNP135. (**B**) Figure B only shows SNPs with a calculated MAF > 0.01 in at least one population from four (CEU, HCB, JPT, YRI).

We identified SNPs either located in human pre-miRNA genes or in their adjacent upstream/downstream 200 bp regions by comparing the coordinates of both SNPs and related miRNAs (Figure 1). There were 12,846 polymorphisms, including 1,940 SNPs in pre-miRNA sequences. An SQL script was written to calculate the SNP density (Figure 2). The SNP density in pre-miRNA regions declined rapidly to 0.43 SNPs/kb if we considered only SNPs having a MAF above 0.01 in four populations (CEU, HCB, JPT, YRI).

### Identifying polymorphisms in miRNA target sites

**Figure 3:**
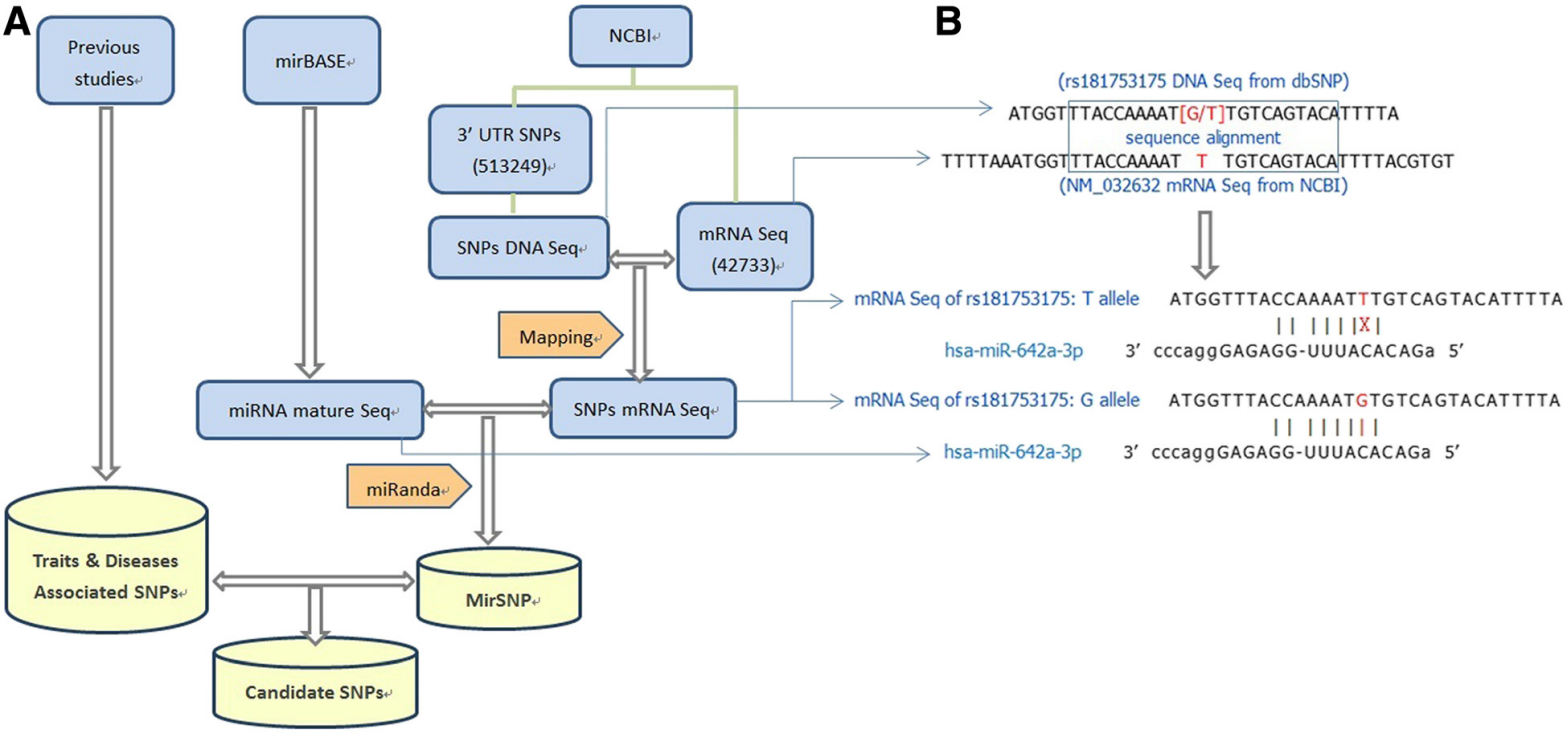
**(A) Workflow depicting the analysis of variations in miRNA-mRNA binding sites.(B) An example of how we determinate a SNP in predicted mRNA target site.**

The method of identifying polymorphisms in mRNAs affecting miRNA-mediated processes is shown in Figure 3. The information as to whether or not a SNP is located in the 3’UTR of an mRNA came from dbSNP135. Only SNPs located in mRNA 3’UTR areas were recorded in the local SQL database. Preforming sequence alignment between 20-bp DNA sequences surrounding 3’UTR SNPs and the corresponding mRNA sequences, variants were mapped onto their mRNA sequences. Subsequently, each SNP in our database had two to four mRNA sequence records corresponding to different alleles. Using the mRNA sequences of SNPs and miRNA mature sequences, we obtained the predicted target results using an miRNA target prediction algorithm, miRanda, which has highest sensitivity among eight tested algorithms [14].

Although there are examples that imperfect 7-nt seed site pairing can be functional, there is overwhelming evidence to support the hypothesis that Watson-Crick pairing to the miRNA seed site is the most important feature for miRNA prediction and function [6,7]. Therefore, we adopted the 7-nt seed region in the miRNA as the major criterion in the miRanda algorithm [15]. In detail, to predict miRNA binding sites, we applied miRanda v3.3a with the default pairing score cutoff of “≥140” and “–strick” command, which only considered stringent 7-nt seed pairing (requiring an uninterrupted match of at least seven nucleotides from the 5’-end of the miRNA).

### Additional information about MirSNP

A requirement for perfect 7-nt seed site pairing improves the reliability of miRNA target prediction, particularly when seed motif is conserved in the UTR regions of whole-genome alignments [16,17]. We downloaded the conservative information of whole-genome alignments (phastCons 46way vertebrates from UCSC ftp site, see URLs) [18] and then added the average value of conservative scores of 7-nt seed motif to our database. The score of mirSVR methodology, a machine learning method for ranking miRNA target sites [19], were also added to the MirSNP database as annotation. However, the data of MirSNP are not identical to that of the mirSVR. Imperfect overlap may traced back to SNPs (the mirSVR methodology didn’t consider the impact of SNPs on miRNA-mRNA bindings), the use of different UTR database and miRNA information. Therefore, not all results in MirSNP should have the score of mirSVR.

SNPs become more important if they have a high frequency or are undergoing positive selection. Therefore, we have added MAF information from dbSNP135 (see URLs) for the SNPs into MirSNP. Based on this information, the results of MirSNP filtered by MAF could be displayed. Analysis of the MAF data revealed that there were 32,822 SNPs located in miRNA-mRNA binding sites with MAFs greater than 0.01 in the four populations. In addition, 122 SNPs in pre-miRNA genes had MAF data and the remaining 1,818 SNPs lacked MAF data.

LD information was obtained for each SNP from the HapMap project [20]. The Phase2 HapMap fileset (see URLs) was downloaded for the four populations and the linkage of the SNPs was calculated using a threshold of *r*^*2*^ greater than 0.8 using the PLINK software [21]. LD and MAF information of SNPs were stored into separate tables in download page (see URLs).

### Database construction

All the useful data were stored in a local MySQL database. We used Django, a web application framework written in Python, to build a user-friendly online website (http://cmbi.bjmu.edu.cn/mirsnp).

## Utility

### The website

**Figure 4:**
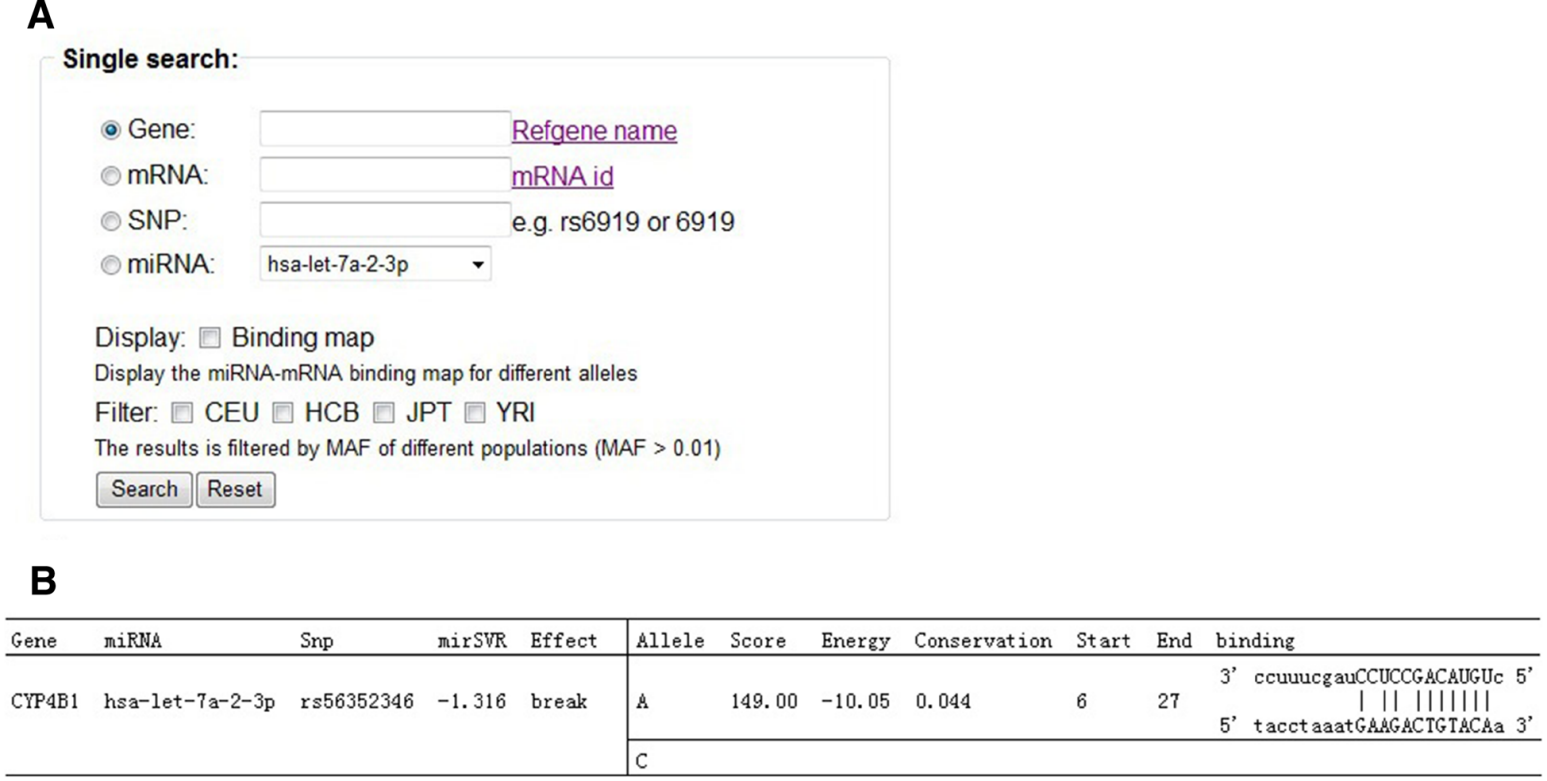
(A) The “Single search” frame of the MirSNP web site. (B) An example of miRNA-related SNP search results.

We obtained over one million records for 414,510 miRNA-related SNPs. These SNPs were classified into four groups, labeled as create, enhance, decrease or break. To display the records in the MirSNP database, we designed a user-friendly MirSNP web site (see URLs) to allowing searching for SNPs in putative miRNA target sites. On the frame “single search” (Figure 4A), the system allows users to search records by entering a RefGene name, mRNA id, SNP RSid or mature miRNA name. All identifiable RefGene names and mRNA ids for this search can be found by clicking the hyperlinks “Refgene name” and “mRNA id” on the page. After pressing the “Search” button, the results are presented on a new page (Figure 4B). The record in Figure 4B shows that the A-allele of rs56352346 may promote the binding of CYP4B1 gene and hsa-let-7a-2-3p and when rs56352346 is the C-allele, the mature miRNA and gene cannot combine (Figure 4B). The specific explanation of each column is provided in the help page. Additionally, on the “single search” frame, users can choose whether to display the binding alignment as well as whether to filter the output by MAF.

**Figure 5:**
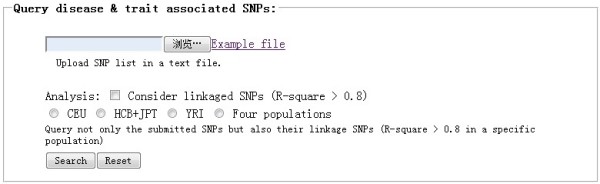
The “Query disease & trait associated SNPs” frame of the MirSNP web site.

We would like to recommend the frame named “Query disease & trait associated SNPs” (Figure 5). This frame permits the search of SNPs from GWAS and eQTL studies. The acceptable input is a list of SNP RSid in a text file. Here, users can query not only the submitted SNPs, but also their linked SNPs (r^2^ > 0.8). For example, searching for associated SNPs in our schizophrenia GWAS data (p < 0.0001) in this frame, four additional SNPs were identified compared to using the “batch search” frame that does not consider linked SNPs.

### The use of MirSNP for brain eQTL data sets

Many studies have implicated the association between SNPs and the expression of their host genes. We speculate that it will be of great significance to combine our miRNA-related SNPs data and eQTL data. We used the MirSNP database for three human brain eQTL data sets, including human cortical samples from 193 individuals [22], cortex samples from 269 individuals [23] and samples obtained from 150 subjects [24]. SNPs located in miRNA-mRNA binding sites could affect the expression of their host genes; therefore, we only considered eQTLs that had a cis-effect on the host genes. Four putative miRNA-related SNPs (rs3087822, rs13042, rs1058381, rs1058398) were selected and they were statistically significant, genome-wide, in the three brain eQTL data (Table 1). Based on our *in silico* analysis, we hypothesize that these SNPs may affect the miRNA mechanism and thus affect the mRNA expression of their host genes in the brain. Further experiments are necessary to confirm our speculation concerning these SNPs.

**Table 1:** Brain eQTLs found in predicted miRNA-mRNA binding sites

**mirSNP**	**Gene**	**Involved miRNAs**	**Allele**	**eQTL**	**Linkage**^**a**^	**p-value (Myers, A.J. et al)**^**b**^	**p-value (Colantuoni, C. et al)**^**c**^	**p-value (Gibbs, J.R. et al)**^**d**^
rs3087822	CRIPT	hsa-miR-200a-3p, hsa-miR-26b-3p, hsa-miR-29b-1-5p, hsa-miR-335-3p	A/G	rs3087822	1	8.45E-05	9.54E-18	3.20E-13
rs13042	FAM82B	hsa-miR-202-5p, hsa-miR-362-5p, hsa-miR-500b, hsa-miR-1914-5p	A/G^e^	rs13042/rs4961193	1/0.864	5.03E-06	8.52E-16	1.30E-08
rs1058381	RABEP1	hsa-miR-4760-3p	A/G	rs1058381/rs1065483	1/0.954	0.00254	7.81E-19	2.89E-08
rs1058398	RABEP1	hsa-miR-134, hsa-miR-3118, hsa-miR-943, hsa-miR-192-3p, hsa-miR-5002-3p	A/G	rs1065483	0.955	0.00254	7.81E-19	3.92E-10

^a^Linkage analysis of predicted miRNA-related SNPs and eQTLs in 90 CEU individuals from HapMap project.

^b^A survey of gene expression of 193 normal human brain tissues (cortex) [22]. The p-values have been adjusted by Bonferroni correction.

^c^A survey of gene expression of 269 normal human brain tissues (prefrontal cortex) [23]. Genome-wide Bonferroni corrected p = 0.05 (2.6e-12).

^d^A survey of gene expression of 150 normal human brain tissues (cerebellum, frontal cortex, pons, and temporal cortex) [24].

^e^Although rs13042 has four allelotypes in MirSNP, only A and G alleles have frequencies in populations.

### The use of MirSNP for a schizophrenia GWAS data set

Our previous GWAS data [25], involving 746 SCZ cases and 1,599 healthy controls, identified a set of 7,705 SNPs having a statistical significance of *p* < 0.01. Here, we conducted a genome-wide analysis for these GWAS SNPs falling within computationally predicted miRNA targets. We combined putative miRNA-related SNPs and GWAS SNPs with SNP id as the key. To increase the range of combination, we used HapMap data and the software Plink to calculate *r*^*2*^ between pairwise SNPs. The GWAS data are from Chinese Han population; therefore, we chose 90 Asian individuals from the HapMap project for LD analyses. A subset of 4,997 SNPs in predicted miRNA target sites were in our GWAS analyses. Hence, we set 1.0 × 10^-5^ (0.05/4997) as the threshold of statistical significance. Three polymorphisms were identified (Table 2). The SNP that showed the strongest association with schizophrenia was found in the *TBC1D15* gene (*p* = 4.0 × 10^-6^ in the Chinese Han population). The *in silico* analysis implied that three SNPs (rs17110432, rs11178988, and rs11178989) in 3’UTR area of *TBC1D15* may affect the miRNA-mRNA binding process. However, further experiments are necessary.

**Table 2:** Putative miRNA-related SNPs associated with schizophrenia

**mirSNP**	**Involved gene**	**MAF**^**a**^	**Involved miRNAs**	**Allele**^**b**^	**SCZ-associated SNP**	**Linkage**^**c**^	**p value**	**OR**
rs11178988	TBC1D15	0.156	hsa-miR-145-3p, hsa-miR-3680-3p, hsa-miR-5689, hsa-miR-1294	C/T	rs17110426	0.945521	4.06E-06	0.6449
rs11178989	TBC1D15	0.156	hsa-miR-4501	A/C	rs17110426	0.945521	4.06E-06	0.6449
rs17110432	TBC1D15	0.167	hsa-miR-1193, hsa-miR-335-3p, hsa-miR-29b-2-5p	A/G	rs17110426	1	4.06E-06	0.6449
rs11544338	FAM117B	0.305	hsa-miR-409-3p, hsa-miR-653	A/C/G/T	rs11544338	1	5.48E-06	0.6511
rs11680951	FAM117B	0.291	hsa-miR-516a-3p, hsa-miR-376a-5p, hsa-miR-1287, hsa-miR-145-3p, hsa-miR-5191, hsa-miR-516b-3p	A/C/G/T	rs11544338	1	5.48E-06	0.6511
rs6058896	DNMT3B	0.078	hsa-miR-30b-5p, hsa-miR-3686, hsa-miR-30c-5p, hsa-miR-4773, hsa-miR-2278, hsa-miR-589-3p	C/T	rs6058894	0.946365	2.31E-05	1.584
rs10484565	TAP2	0.078	hsa-miR-3689b-5p, hsa-miR-3177-5p, hsa-miR-3689a-5p, hsa-miR-3689f, hsa-miR-190b, hsa-miR-3689e, hsa-miR-190a	A/G	rs10484565	1	3.58E-05	1.448
rs670358	CDC42BPG	0.395	hsa-miR-4305, hsa-miR-331-3p, hsa-miR-4638-3p, hsa-miR-4705, hsa-miR-3690, hsa-miR-5195-5p, hsa-miR-4308	A/G	rs670358	1	4.68E-05	0.7677
rs11563929	STEAP2	0.1	hsa-miR-16-2-3p, hsa-miR-195-3p, hsa-miR-3942-5p, hsa-miR-4703-5p, hsa-miR-4766-3p	G/T	rs11563929	1	6.81E-05	0.7281
rs16870907	TAP2	0.047	hsa-miR-4760-3p, hsa-miR-330-3p	C/T	rs16870907	1	8.48E-05	1.589
rs12178	ZBTB34	0.456	hsa-miR-1267, hsa-miR-501-5p, hsa-miR-3613-3p, hsa-miR-653	A/C/G/T	rs12178	1	9.66E-05	1.277

Eleven putative miRNA-related SNPs link to SCZ-GWAS SNPs with a significance of p < 0.0001.

^a^Minor allele frequencies (MAF) are from an HCB population.

^b^Allele reports are from NCBI.

^c^Linkage analysis of putative miRNA-related SNPs and SCZ-associated SNPs in 90 Asian individuals from the HapMap project.

We also overlapped the SNPs around microRNA genes in our GWAS data of schizophrenia. A subset of 108 SNPs around miRNA genes was identified. We found one SNP (rs10173558), which is only 5 bp away from the start site of “hsa-mir-1302-4”, having a statistical significance lower than 0.001. Yuan et al. reported that the highest expression level of miR-1302’ target genes was in the nervous system and the genes were enriched in both synapses and intracellular membrane-bounded organelles [26]. This finding implied a potential relevance of miR-1304 to psychiatric disorders.

## Discussion

In recent years, increasing numbers of databases have been published to aid researchers to explore the impact of SNPs on miRNA targets [27-33]. Some researchers, such as Richardson [31] and Ziebarth [32], have provided links between SNPs in miRNA target sites, cis-acting eQTLs and the results of GWAS. Previous works summarized the characteristics of miRNA-related SNPs and showed the potential of applying such databases in GWAS and eQTL researches. However, these databases may not be suitable for a single GWAS or eQTL data set. Some databases cannot perform batch search for numbers of SNPs and some cannot provide effective miRNA-related information for strongly- associated loci. MirSNP was developed to identify putative miRNA-related SNPs from single data sets of GWAS or eQTL, especially from newly published data sets. First, our analysis covered 513,249 known 3’UTR SNPs based on dbSNP135 and we used highly consistent data sources to avoid data loss while integrating different data. Furthermore, sequence alignments between surrounding DNA sequences of SNPs and the corresponding mRNA sequences were used to map variants into their mRNA sequences. Finally, our web site was designed to directly use GWAS or eQTL data sets in a batch query, particularly considering the linked SNPs in different populations.

**Figure 6:**
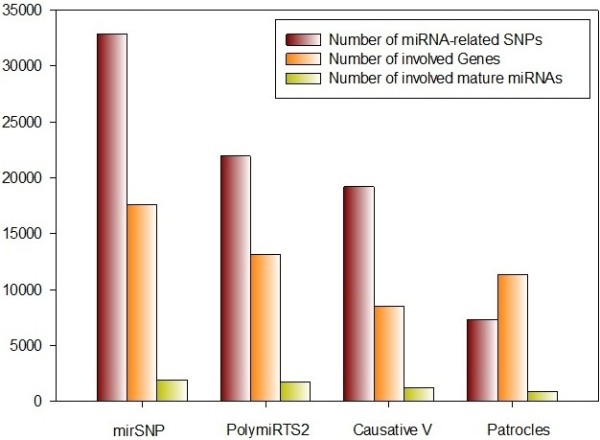
Comparison between MirSNP and three similar databases.

The MirSNP database stores a large number of records of SNPs in predicted miRNA targets sites and we concentrated on providing a convenient search platform so that recent GWAS or eQTL results can be placed on this platform for batch retrieval. In MirSNP, all 3’UTR SNPs stored in dbSNP135 database (513,249) were analyzed. Compared with other databases, MirSNP obtained more results for both common and rare SNPs that might influence miRNA processes. In Table 3 and Figure 6, a comparison between MirSNP and three existing databases (PolymiRTS, Mirsnpscore and Patrocles) are presented. To compare the sensitivity of MirSNP prediction, we identified 13 validated miRNA-related SNPs by literature review: most of the SNPs coming from the table 2 in Sethupathy and Collins [34]. Of the 13 cases (Table 4), nine were identified using MirSNP. The SNPs that not found were either not located in the 3’UTR based on our records (rs34764978, rs13212041) or do not have a perfect 7-nt binding in the seed site (rs2735383, rs9341070). For the other three databases, from thirteen cases, no more than five validated SNPs were identified. Therefore, the MirSNP database could cover majority of validated miRNA-related SNPs.

**Table 3:** Comparison among four databases

	**MirSNP**	**PolymiRTS 2.0**	**Mirsnpscore**^**a**^	**Patrocles**
**Data source**	**NCBI**	**UCSC**	**UCSC**	**UCSC**
**Predicting strategy**	**miRanda**	**TargetScan**		**L motifs**[17]
Results				
miRNA-related SNPs	414510	117037	19513	26578
miRNA-related SNPs with MAF > 0.01	32822	21938	19208	7334
Involved Genes	17569	13129	8495	11314
Involved mature miRNAs	1921	1738	1222	846
Records (SNPs with MAF > 0.01)	121796	78351	69026	10908
Overlap with MirSNP				
Number of SNPs (MAF > 0.01) coinciding with MirSNP		15913	17481	5759

**Table 4:** Experimentally validated miRNA-related SNPs found in MirSNP

**MiRNA**	**Target gene**	**Functional SNP**	**Reported associated disease**	**MirSNP**	**PolymiRTS2**	**Mirsnpscore**	**Patrocles**	**Pubmed id**	**Journal**	**Author**
hsa-mir-629	NBS1	rs2735383	Lung cancer					22114071	Carcinogenesis	Yang L
hsa-mir-184	TNFAIP2	rs8126	Squamous cell carcinoma of the head and neck	✓	✓			21934093	Carcinogenesis	Liu Z
hsa-mir-1827	MYCL1	rs3134615	Small-cell lung cancer	✓		✓	✓	21676885	Cancer Res	Xiong F
hsa-mir-148a	HLA-C	rs67384697	HIV	✓				21499264	Nature	Kulkarni S
hsa-mir-191	MDM4	rs4245739	Ovarian carcinomas	✓		✓		21084273	Cancer Res	Wynendaele J
hsa-mir-125b	BMPR1B	rs1434536	Breast cancer	✓	✓	✓	✓	19738052	Cancer Res	Saetrom P
hsa-mir-510	HTR3E	rs56109847	Diarrhea predominant irritable bowel syndrome	✓	✓			18614545^a^	Hum. Mol. Genet	Kapeller J
hsa-mir-96	HTR1B	rs13212041	Arson or property damage					18283276^a^	Mol. Psychiatry	Jensen KP
hsa-mir-433	FGF20	rs12720208	Parkinson’s disease	✓		✓		18252210^a^	Am. J. Hum. Genet	Wang G
hsa-mir-148/152	HLA-G	rs1063320	Childhood asthma	✓	✓	✓	✓	17847008^a^	Am. J. Hum. Genet	Tan Z
hsa-mir-24	DHFR	rs34764978	Methotrexate resistance					17686970^a^	PNAS	Mishra PJ
hsa-mir-155	AGTR1	rs5186	Hypertension	✓	✓			17668390^a^	Am. J. Hum. Genet	Sethupathy P
hsa-mir-206	ESR1	rs9341070	Breast cancer					17312270^a^	Mol. Endocrinol	Adams BD
hsa-mir-24	SLITRK1	rs193302862	Tourette’s syndrome	✓				16224024^a^	Science	Abelson JF

Some cases are not in MirSNP because the SNPs were not located in the 3’UTR based on our records (rs34764978, rs13212041) or do not have a perfect 7-nt binding in the seed site (rs2735383, rs9341070).

^a^These cases are from Table 2 in Sethupathy and Collins [34].

MiRNAs can downregulate gene expression by two posttranscriptional mechanisms: mRNA cleavage or translational repression. In animals, miRNA is thought to have a repressive effect that influences protein expression, not the mRNA levels. However, it has been estimated that over 80 percent of miRNAs acted to lower mRNA levels, which shows that mRNA destabilization is the primary action mode of miRNAs on target mRNAs [35]. In addition, studies have shown that SNPs in seed-sites region can significantly change the expression of the target mRNA and protein [36-40]. eQTL analysis is an experimental method for exploring the relationship between SNPs and mRNA expression at a high throughput. Genetic variants that create or destroy an miRNA binding site may be the casual cis-acting eQTLs. The combination of our MirSNP database and eQTL data will provide possible explanations for the eQTLs. We have already identified four SNPs in prediected miRNA target sites that were proven to be brain eQTLs in three independent studies. Further experiments are needed to prove that these eQTLs affect gene expression through an miRNA mechanism. On the other hand, using GWAS, enormous numbers of associations between SNPs and diseases have been reported. There are many disease-associated SNPs that function by miRNA regulation. Overlapping miRNA-related SNPs and the existing GWAS data could identify possible biological mechanisms for these disease-associated variations and provide the *in silico* basis for further studies.

Our aim was to merge the MirSNP database with high throughput SNP experimental data. We identified many SNPs in predicted miRNA targets and indicated their potential functions based on sequence algorithms. Unfortunately, the expression information of miRNAs and mRNAs are not supplied in our database. We considered the possibility of an miRNA and mRNA combining, but under the complex mechanism of spatial and temporal expression, we had no idea if the two molecules would encounter each other *in vivo*. Combining MirSNP with additional databases containing expression information, such as miRGator [41], would improve the functionality of the database.

## Conclusions

MirSNP is a database of SNPs in predicted miRNA target sites, based on information from dbSNP135 and mirBASE18. MirSNP is highly sensitive and covers most experiments confirmed SNPs that affect miRNA function. The results suggest that our prediction and data processing are full-scale. MirSNP may be combined with researchers’ own GWAS or eQTL positive data sets to identify the putative miRNA-related SNPs from traits/diseases associated variants. We aim to update the MirSNP database as new versions of mirBASE and dbSNP database become available.

## Availability and requirements

MirSNP is publicly available on the internet at http://cmbi.bjmu.edu.cn/mirsnp.

### URLs

MirSNP (http://cmbi.bjmu.edu.cn/mirsnp)

dbSNP135 (ftp://ftp.ncbi.nih.gov/snp/organisms/human_9606/database/organism_data)

mRNA sequence file (ftp://ftp.ncbi.nih.gov//refseq/H_sapiens/mRNA_Prot/human.rna.fna.gz, released November 15, 2011)

HapMap fileset (http://pngu.mgh.harvard.edu/~purcell/plink/res.shtml)

mirBASE (http://www.mirbase.org/ftp.shtml)

UCSC (ftp://hgdownload.cse.ucsc.edu/goldenPath/hg19/phastCons46way/vertebrate/)

## Abbreviations

3’UTR: 3’ untranslated region; CEU: Samples of Utah residents with Northern and Western European ancestry from the CEPH collection; HCB: Samples of Han Chinese in Beijing; JPT: Samples of Japanese in Tokyo; YRI: Samples of Yoruba people in Ibadan; eQTL: Expression quantitative trait locus; GWAS: Genome-wide association study; LD: Linkage disequilibrium; MAF: Minor allele frequency; miRNA: microRNA; SCZ: Schizophrenia; SNP: Single nucleotide polymorphism; SQL: Structured Query Language.

## Competing interests

The authors declare that they have no competing interests.

## Authors’ contributions

LCX designed and implemented the database, and performed all data processing. LM and LCX designed the web interface. YWH, WLF and ZFQ participated in the analysis of SCZ-associated SNPs and eQTLs. ZD and LTT planned and directed the project. LCX, LTT and YWH drafted the manuscript. All authors have read and approved the manuscript.

## Supplementary Material

Additional file 1: Table S1 Disease-associated SNPs found within microRNA genes. We queried articles for all SNPs located in human pre-miRNA genes and their adjacent upstream/downstream 200 bp regions. 286 articles were found that were published before November, 2011. We reviewed these papers and selected 22 SNPs that were reported to be associated with diseases. ^a^Pubmed ids and SNP information are from dbSNP135. ^b^microRNA positions are from mirBASE18. ^c^The MAF column reports the allele frequency of the minor allele from the four populations where it was the highest. Click here for file
